# Bacteriology

**Published:** 1904-07-16

**Authors:** 


### BACTERIOLOGY.

(*Concluded from page* 263.)

**Food Poisoning and Epidemic Diarrhœa.**—Delepine maintains that epidemic diarrhœa is generally the result of a more widely disseminated, and less massive form of bacterial infection of food. The symptoms of acute food-poisoning and those of epidemic diarrhœa are identical, and there are no points of pathological distinction. Reviewing the bacteriological work done in this field, he mentions the following organisms as being known to excite outbreaks of epidemic diarrhœa:—Varieties of B. Coli, Finckler-Prior spirillum, B. lactis aerogenes, Proteus vulgaris, staphylococci, streptococcus enteritidis, B. enteritidis sporogenes. The slowly-developing gastro-intestinal disturbance with secretory troubles, and paralysis due to the B. botulinus he regards as a distinct disease. Whilst there is no specific bacillus capable of causing epidemic diarrhœa the chief causes of our summer disease are bacilli of the coli group, of which the B. coli communis of Escherich and the B. enteritidis of Gærtner are the two extreme types. Of these two, Gærtner's bacillus is the more virulent, and bacteria of this type are distinguished by the fact that they form no permanent acidity, and do not coagulate milk or change its smell. To produce serious consequences the infection of the milk must be massive, or the food kept for a length of time. Milk is generally infected at the farm or (through vessels) in transit, and the source of infection is primarily fæcal. The addition of preservatives to milk may positively increase the risk; for these, whilst they delay the appearance of acidity and clotting, do not check the growth of bacteria. At summer temperature the bacillus of enteric fever and B. coli and B. enteritidis were found to continue to multiply in milk containing 35 grains, 70 grains, and 140 grains of boracic acid to the gallon. Formalin is added to milk in the proportion of one grain to the gallon, and even with this dilution there is evidence that the health of young animals may be affected, digestive processes delayed, and proteid substances rendered less soluble. The delay in lactic acid fermentation brought about by the addition of the chemical allows the vendor to sell to the consumer a stale and often dirty article.

**Human and Bovine Tuberculosis.**—Neild Cook[8] contributes a useful paper on bovine and human tuberculosis in India. He points out that all authorities agree upon the extreme rarity of bovine tuberculosis in India, and that this is attributable to the fact that cattle live almost entirely in the open. Hindus do not eat the flesh of cow or ox, yet tuberculosis is more common amongst them than amongst the Mahomedans who do partake of beef. Milk is either boiled or converted into ghee before being eaten, and in either case the tubercle bacillus would be destroyed. In spite of this, human tuberculosis is a very prevalent disease, and is almost entirely of the pulmonary type, it being rare even in children to see tuberculosis of the abdominal and cervical lymph-glands. Cook concludes from these facts that Koch's contention that human and bovine tuberculosis are two distinct entities is correct. The question whether cows which react to tuberculin but in whom there is no clinical manifestation of udder disease can secrete tubercle bacilli in the milk is still disputed. Rabinowitsch[9] has always maintained that this is possible and states that the failure to confirm her results has been due to the fact that other observers have inoculated too few animals, or animals, such as the rabbit, that are not highly susceptible. Tubercle bacilli are not always present in such milk and many negative results must be anticipated. The interim report of the Tuberculosis Commission[10] controverts Professor Koch's hypothesis as to the different nature of human and bovine tuberculosis. They have inoculated cows, calves, and heifers with human tuberculous material, the sputum from cases of phthisis, portions of tubercular lung, caseous material from cervical, bronchial, and mesenteric glands, and the tubercular material from joints. Seven out of 20 of these specimens gave rise to acute general tuberculosis in cattle, and all but two of the remainder gave rise to some milder form of tuberculosis which could be further intensified by passage through other animals. In the two remaining cases there resulted only a local tubercular lesion at the site of inoculation. The lesions thus excited in cattle by the inoculation of human tuberculous material differed in no respect from the lesions excited by the inoculation of bovine tubercle, and the Commission regard the two diseases as identical. They see no reason to modify in any way the regulations in force at the present time for the prevention of the spread of infection by meat or milk. Over two hundred cattle have been used for the purposes of the investigation.

[7] Trans. Epidem. Soc. Lond., vol. xxii. [8] Ibid. [9] Zeit. f. Thier. Med. Bd. viii. 1904. [10] Lancet, June 4, 1904.

